# Cellular Mechanisms of Liver Regeneration and Cell-Based Therapies of Liver Diseases

**DOI:** 10.1155/2017/8910821

**Published:** 2017-01-22

**Authors:** Irina V. Kholodenko, Konstantin N. Yarygin

**Affiliations:** Institute of Biomedical Chemistry, Moscow, Russia

## Abstract

The emerging field of regenerative medicine offers innovative methods of cell therapy and tissue/organ engineering as a novel approach to liver disease treatment. The ultimate scientific foundation of both cell therapy of liver diseases and liver tissue and organ engineering is delivered by the in-depth studies of the cellular and molecular mechanisms of liver regeneration. The cellular mechanisms of the homeostatic and injury-induced liver regeneration are unique. Restoration of the mass of liver parenchyma is achieved by compensatory hypertrophy and hyperplasia of the differentiated parenchymal cells, hepatocytes, while expansion and differentiation of the resident stem/progenitor cells play a minor or negligible role. Participation of blood-borne cells of the bone marrow origin in liver parenchyma regeneration has been proven but does not exceed 1-2% of newly formed hepatocytes. Liver regeneration is activated spontaneously after injury and can be further stimulated by cell therapy with hepatocytes, hematopoietic stem cells, or mesenchymal stem cells. Further studies aimed at improving the outcomes of cell therapy of liver diseases are underway. In case of liver failure, transplantation of engineered liver can become the best option in the foreseeable future. Engineering of a transplantable liver or its major part is an enormous challenge, but rapid progress in induced pluripotency, tissue engineering, and bioprinting research shows that it may be doable.

## 1. Introduction

Liver diseases pose a significant problem for national health care systems throughout the world [1–4]. Persisting hepatitis infection, alcoholism, and hereditary liver metabolic disorders are the ultimate cause of growing incidence of acute and chronic liver failure associated with high morbidity and mortality. In case of liver failure clinical approaches currently in use are ineffective with the exception of organ transplantation. While allogeneic liver transplantation is an efficient method, its practical application is curbed by the limited supply of donor organs, immunological side effects, and economic reasons.

The development of alternative methods of treatment of liver pathology is in great demand. The emerging field of regenerative medicine offers novel approaches to liver disease treatment based on a remarkable progress in basic biomedical research during the last 20–30 years. At present, cell therapy (injection or transfusion of cell suspensions) and tissue/organ engineering are the main methods of regenerative medicine studied in the experimental setup and tested clinically. Cell transplantation is aimed at repopulating liver tissue with hepatocytes, to boost the recipient's own liver regeneration capacity and enhance restoration of its structure and function. Compared to organ transplantation or organ/tissue engineering, cell therapy is much less invasive and expensive. On the other hand, organ engineering has the potential to solve the problem of liver donor shortage.

Obviously, the ultimate scientific foundation of both cell therapy of liver diseases and liver tissue and organ engineering should be delivered by the studies of cellular and molecular mechanisms of liver regeneration operating under physiological conditions (homeostatic regeneration), during enhanced functional loading (adaptive regeneration), or after damage caused by disease, poisoning, or trauma (injury-induced regeneration) [5]. Classical textbooks of histology, such as the 9th edition of Ham's Histology [6], used to give the following list of liver cell types: hepatocytes, cholangiocytes (biliary epithelial cells, bile duct epithelium), hepatic macrophages (Kupffer cells), fenestrated endothelium of vascular sinusoids, cellular elements of other blood vessels, Ito cells (stellate cells), stromal fibroblasts, lymphatic vessel cells, lymphocytes and other immune cells, and nerve elements. Later the so-called oval cells were added to the list [7, 8]. Liver hosts a population of stem/progenitor cells, which in rodents includes oval cells [9]. Cell fate experiments suggested that stellate cells can also be the precursors of liver epithelial cells [10, 11].

There are two extensively studied mechanisms of liver regeneration: compensatory hyperplasia of hepatocytes and stem/progenitor cell-mediated regeneration. Molecular and cellular events taking place during compensatory hyperplasia are relatively well characterized, while the alternative regeneration mechanism has not been fully disclosed yet. Except resident liver cells, this role has been attributed to blood-borne cells of the bone marrow origin [12–14]. Stellate cells are an important element of the machinery of liver regeneration being part of liver stem cell niche, supporting regeneration at early stages by secreting growth factors and inducing regeneration arrest after restoration of normal organ mass [15].

Research carried out with animal models showed that methods of regenerative medicine can provide beneficial effects surpassing those delivered by any other therapeutic approach excluding donor liver transplantation and that the mechanisms of those effects involve replacement of damaged cells or tissue and stimulation of the animal's own regenerative potential. Experimental results provided a solid basis for initial clinical research. Limited experience with human patients seems to confirm some of the experimental results.

The scope of this paper is to review the mechanisms of liver regeneration and to describe current approaches aimed at enhancing liver regenerative capacities and at creating new engineered liver tissue and ultimately the whole engineered organ.

## 2. Histological Structure of Liver Tissue

**Figure 1:**
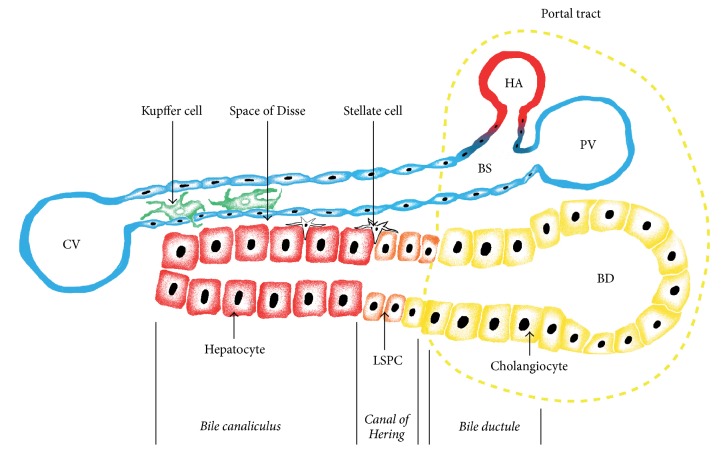
Schematic histological structure of liver tissue. Functional units of liver tissue are formed by trabeculae and accompanying blood sinusoids. Liver tissue gets its afferent blood supply from two sources: hepatic artery and portal vein. Hepatic arterioles (HAs) and the terminal branches of portal vein (PV) merge to form blood sinusoids (BSs) lined with endotheliocytes and drained into the central veins (CVs). In the sinusoids, close to endothelium reside liver macrophages named Kupffer cells. Bile produced by hepatocytes flows in the opposite direction and is discharged into the bile ducts (BDs). Hepatic arterioles, terminal branches of portal vein, and the smallest bile ducts are drawn together forming compact structures called portal tracts shown at the right side of the figure. Liver trabeculae are built of hepatocytes. The inner cavities of trabeculae form canaliculi which are closed at the central ends of the lobules (left side of figure) and while on their way to BD they convert into bile ductules (BDLs) via a transitory zone called the canals of Hering (CH). Bile ductules drained into the bile ducts are lined with cholangiocytes, and the canals of Hering contain LSPCs. Tiny spaces between trabeculae and the endothelium of blood sinusoids are called the spaces of Disse (SD). They participate in the bidirectional traffic of different substances between blood and hepatocytes and contain stellate (Ito) cells.

The structural organization of liver tissue schematically presented in Figure 1 is rather uniform throughout the whole organ and reflects its metabolic and secretory functions [6]. Hepatocytes are quantitatively predominant cells constituting about 80% of total liver mass. They form trabeculae each composed of two rows of cells. Spaces between the hepatocyte rows form biliary canaliculi filled with bile evacuated to the bile ductules through the canals of Hering lined by hepatocytes and bile duct epithelial cells. Space between the trabeculae is occupied by blood sinusoids formed by fenestrated endothelium and lined with liver macrophages named Kupffer cells. The planar space between trabeculae and the fenestrated endothelium providing the maximum contact of hepatocytes with blood is called the space of Disse. Scattered among hepatocytes and contacting the spaces of Disse are Ito cells (liver stellate cells) containing lipids and vitamin A. Besides Kupffer cells, liver hosts other immune cells including resident natural killers (pit cells), conventional NK cells, and dendritic cells.

Liver hosts a pool of cells with combined characteristics of stem cells and progenitor cells [9]. Conventionally, liver stem/progenitor cells (LSPCs) are thought to step in the regeneration process after massive liver tissue necrosis caused by toxic assault or other reasons, when hepatocyte proliferation is constrained. In the literature cells with LSPC characteristics are also called hepatic stem/progenitor cells (HSPCs), liver progenitor cells (LPCs), or hepatic progenitor cells (HPCs). LSPCs reside in the ductules and canals of Hering. Though their role in liver regeneration is uncertain, LSPCs are considered an attractive starting material for the cell therapy of liver diseases and liver tissue engineering [16–18].

Hepatic trabeculae are arranged into lobules where trabeculae radiate from the center occupied by the central vein to the periphery of the lobule. Lobules are separated by connective tissue septae which in humans are very thin. Each lobule is surrounded by six neighboring lobules. Areas where the edges of rectangular faces of three lobules meet form the so-called portal areas containing a portal vein, a hepatic artery, and a bile duct. Blood flows from branches of the portal vein through sinusoids where it gets mixed with blood from branches of the hepatic artery. This mixture drains through the sinusoids into a central vein and further into the terminal branches of the hepatic vein. After collecting blood from its branches, the hepatic vein goes into the inferior vena cava. Metabolism and other properties of hepatocytes depend upon their position at the porto-central axis of a lobule [19]. Normal liver tissue contains relatively small amount of extracellular matrix concentrated primarily in the outer connective tissue capsule of the organ.

## 3. Crucial Role of Hepatocyte Proliferation and Hypertrophy in Liver Regeneration after Partial Hepatectomy

Liver has a remarkable ability to regenerate its mass after injury. Eighty-five years ago Higgins and Anderson [20] were the first to demonstrate that after removal of two-thirds of rat liver the organ regains its initial mass and at this point regrowth is halted. This result has been many times confirmed in experiments with laboratory rodents [21]. In rat and mouse studies restoration of the organ's mass occurred via compensatory hypertrophy and hyperplasia of hepatocytes in the intact lobes, while removed lobes and segments did not regrow. Rodent liver consists of five lobes and three of them can be easily removed without substantial damage to the other two. The remaining two lobes increase their size and restore the organ mass. In mice and rats this takes 5–7 days. In case of partial hepatectomy, functional regeneration is not accompanied by full anatomical regeneration suggesting that liver regeneration induced by the removal of its part is guided by functional impairment. Importantly, organ regeneration occurring after living-donor liver transplantation in humans seems to follow a similar route. Indeed, after the operation both parts of donor's liver (that remaining in donor's body and the transplanted one) grow to restore normal organ size to serve both individuals [22].

Regenerative response to partial hepatectomy involves numerous coordinated events occurring at the molecular, cellular, and tissue levels. In mice and rats the removal of three liver lobes leads to immediate changes of hepatocyte gene expression pattern, activation of numerous transcription factors and receptors, and secretion of a number of growth promoting signal molecules into liver parenchyma and circulation [23, 24]. Apparently, liver regeneration after partial hepatectomy is a result of hepatocyte proliferation and hypertrophy but does not involve proliferation and hepatogenic differentiation of LSPCs [21, 23]. Liver regeneration combines hepatocyte hypertrophy and hyperplasia. Hepatocyte hypertrophy starts within hours after partial hepatectomy and is followed by hepatocyte hyperplasia. Full functional restoration after injury should involve restitution of all functions of normal liver including control of blood sugar levels, production of albumin, blood clotting factors and other vital proteins, bile secretion, and neutralization of poisonous substances. In rodents, time required for functional restoration depends upon experimental conditions. In humans, among factors affecting restoration time most are the extent of liver damage, diseases of liver parenchyma, age, and portal pressure [25–27].

In rat and mouse models, DNA synthesis begins 12–16 hours after partial hepatectomy and reaches its maximum after 24–48 hours [21]. The initial mass of liver is restored in about 3–7 days. At this stage the histology of regenerated liver tissue is still very different from normal, hepatocyte propagation slows down, and new blood vessel formation begins [21]. Ito cells secrete beta platelet-derived growth factor (*β*-PDGFR), thus contributing to hepatocyte proliferation arrest [28]. Normal liver histology and functions are restored within 8–10 days.

The relative input of hepatocyte proliferation and hypertrophy in liver size restoration after partial hepatectomy has been extensively studied. If there were no hypertrophy, it would take around 1.6 divisions of an average hepatocyte to regain the organ size after removal of 70% of its mass. However, hypertrophy of hepatocytes after partial hepatectomy is well documented, and it is well known that there are many binuclear hepatocytes in adult liver and their number decreases during posttraumatic regeneration [29, 30]. Using a specially developed method of cell size and ploidy determination, Miyaoka et al. [31] showed that in mice cellular hypertrophy makes the first contribution to liver mass restoration. In their experiments regeneration after removal of 30% of liver was achieved solely by hypertrophy without cell division, while after 70% hepatectomy hypertrophy preceded proliferation. Some of the hepatocytes were undergoing mitosis without cytokinesis and remained binuclear and diploid.

While the crucial role of hepatocyte proliferation and polyploidy in partial hepatectomy-induced regeneration has been repeatedly confirmed, the origin of new hepatocytes in regenerating liver is still a matter of discussion. It has been suggested that some of them descend not from other hepatocytes but from LSPCs or even from blood-borne cells. Early publications provided evidence supporting the predominant role of differentiated hepatocytes, while later many researchers were supporting the “streaming liver hypothesis” claiming constant proliferation of LSPCs during liver homeostasis and after enhancement of the regeneration process after injury [32]. However, other researchers applying improved methods of genetic cell lineage tracing provided additional evidence confirming the crucial role of proliferation of differentiated hepatocytes in homeostatic conditions and after partial hepatectomy. Malato et al. [33] designed an approach ensuring stable expression of the enhanced yellow fluorescent protein in adult murine hepatocytes, making it possible to trace the fate of cells over a period of time. This approach delivered experimental data proving that under normal homeostatic conditions adult differentiated hepatocytes are the source of all new hepatocytes. Moreover, the majority (~99%) of new hepatocytes emerging during partial hepatectomy-induced regeneration also originate from differentiated hepatocytes. Therefore, hepatocytes may be regarded as committed unipotent cells reproducing themselves as suggested by Zhang et al. [34], while there seems to be no place remaining for LSPCs in homeostatic murine liver regeneration and its regeneration after partial hepatectomy. Data concerning the relative impact of hepatocytes and LSPCs in liver regeneration after other types of hepatic injuries will be analyzed in the following section.

Initiation of regeneration after partial hepatectomy may be associated with hemodynamic changes. Surgical removal of two-thirds of liver results in approximately threefold increase of portal pressure [35]. This induces proliferation of several liver cell types: hepatocytes, stellate cells, bile duct epithelium, hepatic macrophages (Kupffer cells), and fenestrated endothelium of vascular sinusoids. Hepatocytes are the first cell type starting DNA synthesis after partial hepatectomy. Hepatocytes remaining in place after the removal of two-thirds of liver undergo one cycle of DNA synthesis yielding reconstitution of 60% of hepatocyte mass [36]. A fraction of hepatocytes enters additional rounds of DNA synthesis ensuring full recovery of liver parenchyma. There is an increase of the number of apoptotic cells at the end of DNA synthesis period reflecting correction of excessive regenerative response [37]. Hepatocyte proliferation starts at portal areas containing a portal vein, a hepatic artery, and a bile duct and proceeds in the direction of the central vein [38, 39]. Hepatocytes surrounding the central vein are the last to replicate [40]. Proliferation of bile duct epithelium starts later than hepatocyte proliferation. It begins at days 2-3 and ends at days 4-5 after partial hepatectomy [40]. The timing of hepatocyte mitosis during liver regeneration is controlled by circadian clock [41, 42]. Some authors found as many as four waves of hepatocyte replication [42].

Compared to other organs, liver contains elevated numbers of resident macrophages (Kupffer cells). Meijer et al. [43] demonstrated the participation of Kupffer cells and migrating monocyte-derived macrophages in liver regeneration after partial hepatectomy. Liposomal clodronate-induced macrophage ablation resulted in slowing down induced hepatocyte proliferation and, as a result, liver inability to fully restore its mass, suggesting an important role of macrophages-produced cytokines and growth factors in the initiation of hepatocyte proliferation. Wnt ligands are probably most critical for switching on hepatocyte proliferation [44, 45].

It should be stressed that microenvironment including cells and extracellular matrix plays a substantial role in the maintenance of liver tissue homeostasis and in regenerative response [23, 46]. Ding et al. [47] proved an important role of endothelium in hepatocyte proliferation support and reconstruction of vascular net in the regenerating liver.

## 4. Evidence for and against the Existence of Alternative, LSPCs-Dependent Mechanisms of Liver Regeneration

During the last three decades growing number of publications described liver regeneration mechanisms activated in case of hepatocyte proliferation blockage. Here we call those mechanisms “alternative” in order to stress their distinction from “classical” regeneration mechanisms implicated in liver regrowth after partial hepatectomy and involving compensatory hypertrophy and hyperplasia of hepatocytes. Instead of adult hepatocytes, alternate liver regeneration mechanisms are supposed to involve LSPCs capable of differentiation into hepatocytes, oval cells (considered LSPCs by many researchers), and, to a lesser extent, Ito (stellate) cells [48].

Historically, rat oval cells were the first described liver cell type with stem/progenitor properties. It has been shown that partial hepatectomy combined with DNA damage results in proliferation of certain cells in the terminal branches of the biliary tree [49]. Lineage tracing with ^3^H-thymidine indicated that these propagating “oval cells” can undergo hepatogenic differentiation thus participating in the replenishment of liver parenchyma. From the very start there were two competing point of views—one regarding oval cells as hepatogenic progenitors and the other claiming that oval cells are dedifferentiated hepatocytes. Partisans of the first point of view maintained that oval cells serve as an emergency tissue compartment providing material for restoration of liver parenchymal cells and can be regarded as LSPCs. Really, it was demonstrated that in rodents a number of hepatic poisons including dipin, retrorsine, or galactosamine reduce the replicative activity of the majority of hepatocytes and induce oval cells to propagate and regrow liver parenchyma [50, 51]. In humans, acute liver damage or chronic liver disease, such as late stage cirrhosis, provokes activation of progenitor cells [52]. Like oval cells, human progenitor cells or LSPCs reside in the canals of Hering [53, 54]. A clinical study carried out with biopsies taken from patients with massive hepatic necrosis occurring after partial liver transplantation demonstrated the crucial role of LSPCs in the parenchyma regrowth after acute liver failure [55]. Significant role of LSPCs in liver regeneration got further experimental and clinical support. For instance, positive outcomes after acetaminophen-induced liver damage seem to be directly correlated with serum alfa-fetoprotein level [56]. Since alfa-fetoprotein is produced mainly by resident progenitor cells, its enhanced production may reflect their active proliferation. Clinical significance of LSPC propagation in late stage cirrhosis patients is obscured by the lack of restoration of hepatocyte numbers and functional recovery [57, 58]. In this case weak proliferation of LSPCs was ascribed to hepatocyte replicative senescence and exhaustion of their proliferative potential.

At present, the concept of LSPC-dependent liver regeneration mechanisms lives on almost in its original form insisting that LSPCs residing in the terminal branches of biliary tree become a major source of newly generated parenchymal cells when standard hepatocyte-dependent regeneration is compromised due to irreversible hepatocyte damage [59]. Conversely, in case of biliary epithelial cells failure to proliferate hepatocytes turns out to be the source of facultative stem/progenitor cells for biliary epithelium. It has been claimed that LSPCs possess high proliferative potential, express bile duct epithelial cell markers, and are able to differentiate into both hepatocytes and bile duct epithelial cells in vitro (reviewed in [60]). However, no specific LSPC markers were detected as yet. LSPC differentiation seems to be driven by the activity of certain genes and a unique combination of growth factors. Crucially important genes include Leucine-rich repeat-containing G-protein coupled receptor 5 (LGR5) [61, 62] and the cytokine tumor necrosis factor-like weak inducer of apoptosis (TWEAK), a member of the tumor necrosis factor (TNF) superfamily [63]. In addition, significant role belongs to known mitogenic factors, such as HGF, epidermal growth factor (EGF), TGF-*α*, and fibroblast growth factors 1 and 2 (FGF1 and FGF2) [64, 65].

In normal human liver cells displaying mixed biliary epithelium/hepatocyte differentiation potential are found in distal parts of the canals of Hering where bile duct epithelium is in close proximity of hepatocytes [66]. Studies in rats and mice reported transdifferentiation of hepatocytes into bile duct epithelial cells under circumstances preventing renewal of the biliary epithelium [67–69]. Most probably, this process involves periportal hepatocytes, a distinct subpopulation of hepatocytes originating from the ductal plate [70, 71]. During embryogenesis both hepatocytes and cholangiocytes descend from common precursor cells known as hepatoblasts. Separation of the hepatocyte and cholangiocyte lineages occurs rather late in rodent embryogenesis and in the middle of the second trimester in humans [72–74]. Therefore, transdifferentiation of bile duct cells into hepatocytes and vice versa really looks quite natural and those two liver cell types are likely to operate as each other's conditional stem/progenitor cells [59, 67]. In humans this mechanism of regeneration is probably activated in fulminant liver failure. In fulminant hepatitis the histological examination of liver tissue reveals clusters of parenchyma regeneration consisting of cells displaying features of differentiation into both hepatocytes and cholangiocytes. In addition, in most cases there is a vast proliferation of bile duct cells starting to express hepatocyte biomarkers [74–76]. Clinical data, as well as the results of the model experiments with laboratory rodents, suggest that in fulminant liver failure hepatocytes are regenerated through cholangiocyte transdifferentiation [59].

All these and many other results accumulated over the years are entirely consistent with the idea of facultative LSPCs serving as a source of hepatocytes after certain hepatic injuries. However, the concept of LSPCs playing a substantial role in liver regeneration was almost destroyed by several papers published in 2014. Four research groups applied innovative lineage tracing methods to determine progenitors of new hepatocytes emerging in the course of regeneration in standard murine liver damage models. Schaub and coauthors [77] using cytokeratin-19 (CK19) lineage tracing for LSPC progeny and high-efficiency hepatocyte marking demonstrated that, in the model of chronic liver injury caused by a special choline-deficient, ethionine-supplemented (CDE) diet regeneration did not involve LSPCs and all new hepatocytes stemmed from hepatocytes. Cell fate tracing utilizing of stellate (Ito) cell progeny in Pdgfrb-cre mice demonstrated that no hepatocytes emerged from those cells as well. This evidence was in line with data presented by Yanger and coauthors [78] showing that independent of the kind of injury preexisting hepatocytes and not LSPCs or oval cells are the main if not the only source of hepatocytes newly generated during adult liver regeneration. The experiments were carried out using four different mouse oval cell injury regimens and three distinct methodological approaches. The first method based on the usage of a bile-duct-specific, tamoxifen-inducible cre-line failed to demonstrate any CK19-marked hepatocytes in any of four injury models. High-efficiency labeling of differentiated hepatocytes combined with oval cell damage and subsequent quantification of unlabeled hepatocytes (2nd method) gave no sign of LSPCs different from hepatocytes. Finally, the third method using nucleoside analogs to track rapidly dividing cells showed no rapidly proliferating oval cells undergoing hepatogenic differentiation. Furthermore, cell fate studies in mice employing biliary cell tracers Hnf1*β* [79] and ductal transcription factor Sox9 [80] also demonstrated negligible involvement of oval cells/LPSCs in postinjury liver regeneration. Together, four described publications established primary role of hepatocytes in homeostatic and especially postinjury liver self-renewal and strongly argued against participation of oval cells/LSPCs in liver parenchyma renewal or replenishment. Hepatocytes were shown to be able to replicate even in case of very severe liver damage. Soon afterwards it was shown that in a mouse chronic liver damage model new hepatocytes originated from the so-called hybrid hepatocytes, a subpopulation of periportal hepatocytes present in intact liver and capable of rapid proliferation [81]. Hybrid hepatocytes are characterized by low level of Sox9 expression and normal expression of hepatocyte nuclear factor 4*α* (HNF4*α*). They express genes specific for hepatocytes and some cholangiocyte-specific genes.

The described lineage tracing experiments clearly changed the previously adopted model of liver regeneration. However, there are doubts concerning generalization of those findings. Accurate cell fate experiments were performed only in mice and it is not yet clear if other species, including rats and humans, also lack LSPCs with in vivo hepatocyte-replenishing capacity. Besides, despite lack of the proof of the in vivo hepatogenic differentiation of LSPCs, they certainly can give rise to hepatocyte-like cells in vitro [48]. Research in this field is ongoing and there is a chance that even in mice a role for oval cells/LSPCs in regeneration will be found. Recently, CD45-TER119-CD31-EpCAM-ICAM-1+ resident progenitor cells, distinct from conventional oval cell-like LSPCs, were described [82]. Those cells were first found in late murine fetuses and postnatal puppies, reach peak numbers by the 4th week after birth, and persist throughout life. They were shown to differentiate into mature hepatocytes in vitro. Upon transplantation they participate in the recipient's liver repopulation after partial hepatectomy combined with retrorsine treatment or after treatment with oncostatin M. They also contribute to cellular turnover in normal healthy mouse liver, that is, participate in homeostatic organ regeneration.

## 5. Involvement of Extrahepatic Stem/Progenitor Cells in Liver Regeneration

Certain extrahepatic cells including hematopoietic stem cells (HSCs) and mesenchymal stem cells (MSCs) of bone marrow origin can be induced to differentiate into liver cells in vitro. There are several lines of evidence suggesting that differentiation of bone marrow HSC or MSC into cells of hepatic lineages may also occur in vivo in physiological conditions and after liver injury. Bone marrow HSC and MSC can easily reach liver through circulation. In embryogenesis the hematopoietic system and liver closely interact [83]. Fetal liver is an important source of HSC [84]. Moreover, HSCs persist in liver tissue throughout adulthood [85]. HSCs were shown to undergo hepatogenic differentiation and to populate liver after intravenous transplantation [34, 86, 87]. Similar data were reported by research groups working with MSC, another major bone marrow stem cell type [34, 88, 89] (Table 1).

It has been noticed that oval cells of adult liver express Thy-1, a surface marker routinely used together with CD34 to specifically identify HSC [90]. Hepatocyte proliferation blocker 2-acethylaminofluorene combined with liver injury to induce oval cell proliferation in rats after cross-sex bone marrow transplantation induced appearance of oval cells bearing the transplanted bone marrow genetic marker, Y chromosome [90]. In a similar study, female mice after cross-sex bone marrow transplantation obtained over 2% hepatocytes with Y chromosome [91]. Interestingly, relatively high levels of liver repopulation with donor-derived hepatocytes in the cited reports were attributed to the blockage of resident hepatocyte proliferation with irradiation [92]. It was reported that in mice HSC represent the main if not unique type of liver-repopulating bone marrow cells [86]. Therefore, data from animal experiments suggest the possibility of participation of HSC and probably other bone marrow cells in liver regeneration. The extent of their participation in humans remains obscure. However, circumstantial evidence suggests that it can take place and play a role in regeneration [14, 91]. Interpretation of experimental and clinical data in this field is difficult, because of the complexity of the involved cellular mechanisms. Some researchers failed to find the proof of participation of bone marrow cells in hepatocyte generation in the course of mouse liver regeneration after partial hepatectomy [93]. Bone marrow-derived hepatocytes usually constitute less than 1% of total hepatocyte population of transplanted human liver and sometimes are not present at all (data reviewed in [23]).

It has been established that only a fraction of HSC participates in hepatocyte replenishment. Initially, it was shown that in a FAH−/− (fumarylacetoacetate hydrolase-deficient) mouse model only (c-Kit^high^Thy^low^Lin-Sca1+)-HSC, but not c-Kit-Sca1− or lineage-positive (Lin+) cells, differentiated into hepatocyte-like cells [86]. Other research groups either confirmed these results or determined different HSC phenotypes [94, 95]. In a recent publication Oh et al. [96] reported isolation of endodermal precursor cells from the subpopulation of Lin-Sca1+ cells of murine HSC. Only those endodermal precursor cells participated in liver repopulation with hepatocytes in two models of liver damage. This finding is in accordance with the suggestion that bone marrow contains multipotent precursors of the parenchymal cells of different tissues [97].

It has been proposed that bone marrow-derived hepatocytes are generated by the fusion of HSC with liver hepatocytes rather than by direct transdifferentiation of HSC into hepatocytes [98, 99]. Though this hypothesis has been disputed [94], it is still getting support in some recent publications. Using fumarylacetoacetate hydrolase-deficient (FAH−/−) mice as a model of liver failure, Li et al. [100] demonstrated that bone marrow-derived hepatocytes can be generated by fusion of BM-derived CD11b+F4/80+ myelomonocytes with resident FAH−/− hepatocytes.

As suggested by Prockop [12], bone marrow MSCs provide a pool of stem cells for nonhematopoietic tissues including liver tissue. Like HSC, MSCs are a very heterogeneous cell population. It has been shown that only a tiny fraction of bone marrow MSC, the so-called multilineage-differentiating stress-enduring (Muse) cells, participate in liver regeneration [101]. After injection of green fluorescent protein-labeled human bone marrow Muse cells into partially hepatectomized immunodeficient mice, immunohistochemistry, in situ hybridization, and species-specific polymerase chain reaction revealed their integration into the liver tissue during the early regeneration phase [102]. Integrated human cells expressed liver progenitor markers and afterwards differentiated into the following liver cell types: hepatocytes (≈74.3% of GFP-positive integrated Muse cells), cholangiocytes (≈17.7%), sinusoidal endothelial cells (≈2.0%), and Kupffer cells (≈6.0%). These data correlated with the results of genotyping of 20 human liver transplants by short tandem repeats which revealed the presence of recipient or chimeric genotypes in hepatocytes (6 of 17, 35.3%), sinusoidal cells (18 of 18, 100%), cholangiocytes (15 of 17, 88.2%), and cells in the periportal areas (7 of 8, 87.5%) [102].

Other research groups characterized different bone marrow MSC culture subpopulations capable of multilineage differentiation, including the so-called multipotent adult progenitor cells [103] and very small embryonic-like stem cells [104]. Still different subpopulations able to differentiate into liver cells and other endoderm derivatives were found in MSC isolated from other sources.

It is noteworthy that bone marrow cells participate in the regeneration of liver endothelium. Endothelial cells of bone marrow origin can comprise more than 20% of replenished liver endothelial cells after liver transplantation in humans or after partial hepatectomy in rodents [93].

Data concerning the participation of the blood-borne bone marrow cells in the regeneration of liver parenchyma are of prime importance for the development of cell and the methods of cell therapy of liver diseases.

## 6. Cell-Based Technologies in Hepatology

Most conventional approaches of modern medicine help to alleviate disease symptoms by repairing biochemical or anatomical abnormalities or substituting missing and damaged parts with prostheses. They do not attempt to mobilize the curative capacities of the patient's own body. Regenerative medicine is a rapidly developing discipline specifically addressing this issue [105]. Regenerative medicine also attempts to directly repopulate damaged tissues with transplanted cells and to substitute malfunctioning organs using tissue/organ engineering techniques. Application of the methods of regenerative medicine in hepatology holds promise to deliver beneficial outcomes surpassing those provided by other therapeutic approaches [106, 107].

At present, the majority of the technologies of regenerative medicine are based on the use of live cells. Other tactics, such as stimulation of regeneration with medicines [108] or usage of the components of cell secretome instead of whole intact cells [109], are just emerging. There are two basic approaches to clinical application of cell-based technologies in hepatology: cell therapy and tissue/organ engineering. Both are at early stages of their development and much more preclinical and clinical research is needed before efficient and safe therapies will be available. In addition, present day regenerative hepatology only partly relies on the achievements in basic liver regeneration research. However, huge investment of effort and resources taking place at present makes ultimate success quite likely.

**Figure 2:**
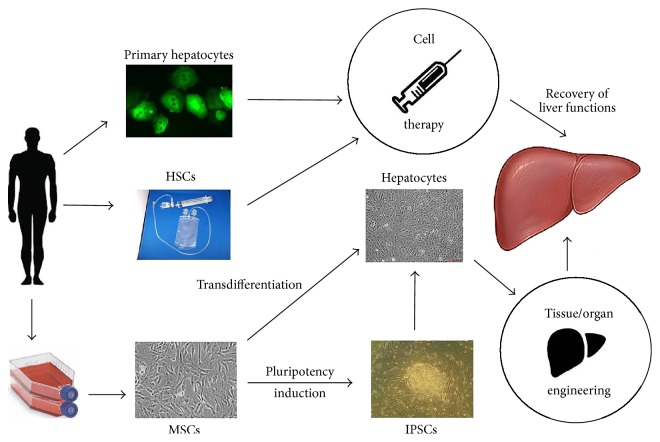
Methods of regenerative medicine for the therapy of liver diseases. Hepatocytes, hematopoietic stem cells (HSCs), and mesenchymal stromal cells (MSCs) are the three cell types commonly used as starting material for the design of cell-based therapies of liver diseases and for liver tissue/organ engineering. Primary hepatocytes isolated from liver biopsies and HSCs isolated from bone marrow or blood, are used for cell therapy after minimum in vitro processing. HSCs can be also expanded in culture (not shown) before transplantation or induced to pluripotency and utilized in cell therapy and tissue/organ engineering applications after hepatogenic differentiation. Chances are that HSCs can be directly transdifferentiated into hepatocytes. MSCs after isolation are in most cases extensively expanded. MSC cultures are then either used for transplantation or transformed into hepatocytes or other liver cells via iPSCs or by direct transdifferentiation. MSC-derived differentiated liver cells are used in cell therapy and tissue/organ engineering applications.

Cell therapy involves transplantation of the suspensions of cells via different routes, most commonly intravenously. It is a relatively inexpensive method with a potential to provide excellent results in some cases. The rationale of cell therapy of liver diseases is twofold. First, some cells, such as autologous hepatocytes, can directly repopulate damaged liver tissue. It is also possible that certain types of cells can transdifferentiate into liver cells after homing within liver tissue. Second, transplanted cells produce beneficial effects, such as stimulation of tissue regeneration, by paracrine secretion of balanced combinations of cytokines, chemokines, growth factors, and noncoding RNAs affecting resident progenitors, extrahepatic stem cells, and other targets (Figure 2).

Tissue/organ engineering encompasses ex vivo assembly of liver tissue, a liver lobe, or whole liver with subsequent orthotopic implantation. Tissue/organ engineering can include a transitory stage of heterotopic implantation of tissue and its growth in vivo. Liver tissue/organ engineering using autologous or allogeneic cells can solve the problem of donor liver shortage. Engineering of whole human liver or its lobe is not just a very challenging but also a very expensive enterprise. The latter is particularly true in case of engineering of an immune conflict-safe liver from autologous cells. Fortunately, recent scientific developments offer clues for scaling up the tissue/organ manufacturing process by the use of immunologically neutral allogeneic cells. In this case organs intended for transplantation can be manufactured using relatively cheap large scale industrial technologies.

### 6.1. Cell Therapy with Hepatocytes

Since compensatory hyperplasia of differentiated adult hepatocytes is the principal way of liver regeneration after injury, repopulation of damaged liver tissue with transplanted hepatocytes or their progeny looks logical. Transplantation of primary (noncultivated) hepatocytes via portal vein proved effective in some animal liver disease models (reviewed in [110, 111]) and treatment of human liver metabolic and genetic disorders like hepatolenticular degeneration (Wilson-Konovalov disease), tyrosinemia, Crigler–Najjar syndrome, urea cycle disorders, severe dyslipidemia, and others (reviewed in [112, 113]). Though homing within liver tissue and participation in its repopulation are thought to be the main mechanism underlying the effects of primary hepatocytes transplantation, stimulation of tissue regeneration through paracrine action is also involved. Unfortunately, accumulated clinical experience is insufficient to define the optimum quantity of infused cells and number of transplantations and intervals between transplantations, as well as the extent and duration of immunosuppression needed. Moreover, transplantation of human primary hepatocytes had virtually no effect in acute liver failure or chronic liver disease [114, 115]. Because of drastic shortage of donor organs, human hepatocytes intended for cell therapy are harvested from livers unsuitable for organ transplantation and, accordingly, are inferior in quality. For that reason, uncertain efficacy or lack of efficacy of primary human hepatocytes transplantation is probably due to poor quality of infused cells.

There were attempts to use primary fetal hepatocytes or hepatoma cell lines instead of adult primary hepatocytes [114, 115]. Cell therapy utilizing those kinds of primary hepatocyte-like cells showed limited efficacy probably because of limited functionality of immature transplanted cells. In addition, fetal material can cause formation of teratomas, while transformed cell lines are thought to have oncogenic potential. A number of research groups reported effective engraftment of xenogeneic hepatocytes into the liver tissue and suggested transplantation of porcine hepatocytes into humans [116, 117]. However, due to safety concerns this option will have hard time getting through the regulation hurdles.

Despite many difficulties, further attempts to improve the methods of ex vivo expansion of human hepatocytes isolated from adult livers, while preserving their metabolic functions, engraftment properties, and in vivo proliferation potential are underway. There is progress in inventing approaches to viable hepatocytes extraction from cadaveric [118] and steatotic [119] livers by adding antioxidants and other beneficial supplements to the perfusion solution. Recently, Levy et al. [120] reported successful oncostatin M-dependent expansion of primary human hepatocytes by inducing low expression of the human papilloma virus genes E6 and E7 coupled with inhibition of epithelial-to-mesenchymal transition. Huch et al. [121] showed that adult bile duct-derived bipotent progenitor cells from human liver can be expanded as epithelial organoids in vitro and then differentiated into functional hepatocytes in vitro and in vivo.

Luckily, progress in cell biology offers new opportunities and alternative ways to generate human hepatocytes, including allogeneic hepatocytes for immunosuppression-free transplantation. Reprogramming of somatic cells into induced pluripotent stem cells (iPSCs) introduced by Yamanaka group [122] presented a unique opportunity to obtain autologous pluripotent cells for cell therapy and tissue engineering. These cells are pluripotent cells similar to embryonic stem cells and can be converted into any differentiated cell type using methods similar to those developed for embryonic stem cells. Reports of successful hepatogenic differentiation of human iPSCs carried out in three or four stages [123–125] were published soon after introduction of the technology of pluripotency induction. Production of hepatocytes via iPSC stage has been successfully scaled up [126, 127]. Methods of large scale production of immunologically neutral allogeneic human hepatocytes from iPSCs will be described in more detail further, in the Liver Organ/Tissue Engineering.

In vitro hepatogenic differentiation of iPSCs yields just partially differentiated hepatocyte-like cells. To undergo full differentiation they need proper microenvironment which is difficult to reproduce ex vivo. However, transplanted cells are likely to get all necessary environmental differentiation stimuli after homing in liver tissue.

### 6.2. Cell Therapy with Liver Stem/Progenitor Cells

After hepatocytes, LSPCs appear to be the most convenient candidates among all liver cell types for restoration of the organ parenchyma and tissue engineering. Indeed, the ability of LSPCs to differentiate into hepatocytes and bile duct cells in vitro has been well documented (reviewed in [60]). Accordingly, it is likely that after transplantation part of LSPCs will undergo homing in the recipient's liver and differentiate into hepatocytes and bile duct cells. Successful in vitro expansion of mouse [61] or rat [128] LSPCs, their engraftment into the liver tissue, and hepatogenic differentiation after transplantation were reported. Efficient expansion of human LSPCs in culture was described as well [62]. Further research is necessary to clarify LSPCs' curative potential.

Liver tissue response to different stimuli and to damage includes orchestrated reaction of all resident cell types. Efficacy of the methods of cell therapy in hepatology will improve parallel to increase of our knowledge about the complexity of interactions of different liver tissue compartments.

### 6.3. Cell Therapy with Hematopoietic Stem Cells

Liver is the site of homing of cells of extrahepatic origin, such as HSCs, stromal cells, and immune cells, suggesting a role for blood-borne cells, including stem cells, in liver regeneration. The next two sections are devoted to cell therapy with HSCs and MSCs.

The scientific basis for cell therapy of liver diseases with HSC transplantation is twofold. First, blood-borne bone marrow-derived HSCs allegedly participate in liver regeneration [8, 86, 129]. Second, in animal models of liver malfunction infusion of HSC improves the outcomes [34, 86, 87]. Therapy with macrophages differentiating from HSCs improves outcomes of liver fibrosis in mice [130]. It has been established that transplantation of hematopoietic cells [131] or their derivatives such as macrophages (analyzed in [132]) alleviates symptoms of human liver diseases as well. The extent of substitution of lost or damaged hepatocytes by differentiating HSC does not exceed 1-2% of newly emerged hepatocytes [129], suggesting that liver tissue regeneration and repopulation after HSCs infusion are essentially induced through paracrine action of transplanted cells. It is possible, though, that the proportion of new hepatocytes differentiating from HSCs can be increased by using the subpopulation of pluripotent cells present in the bone marrow HSC population [94–97]. However, because of the danger of teratoma formation, more animal research is needed before human tests are allowed.

HSC transplantation, especially repeated procedures, can cause adverse immunological reactions and may require immunosuppressive therapy. Adverse effects can be avoided utilizing immunologically neutral allogeneic human cells from cord blood or prepared from iPSCs as described in the Liver Engineering.

### 6.4. Cell Therapy with Mesenchymal Stem Cells

The rationale for MSC-based therapy is similar to that for HSC-based therapy. Prockop [12] was the first to suggest that bone marrow MSCs contain subpopulations of cells participating in regeneration of the parenchyma of different organs including liver. Later, several research groups reported isolation of cells capable of multilineage differentiation from bone marrow-derived MSC cultures, as well as MSC cultures isolated from other tissues. Cells characterized by each group proved to display somewhat unique properties and got different names. The following types of MSC subpopulations received most attention from the scientific community: “multipotent adult progenitor cells” (MAPC) [103], “very small embryonic-like stem cells” (VSEL cells) [104], and “multilineage-differentiating stress-enduring” (Muse) cells [101]. Cells of each of those types can be induced to differentiate not only into mesoderm, but also into ectoderm and endoderm derivatives and are able to home in the parenchyma of internal organs. Existing experimental and clinical data demonstrate considerable involvement of Muse cells in liver repopulation after injury [101, 102]. Specifically, in immunocompromised mice, transplanted human Muse cells integrate into the liver tissue and differentiate in vivo into hepatocytes, cholangiocytes, sinusoidal endothelial cells, and Kupffer cells, producing human proteins [102].

In addition to being directly involved in repopulation of damaged liver tissue, MSCs are also able to stimulate regeneration via paracrine secretion (reviewed in [133, 134]). In accordance with everything written above in this section, transplantation of MSC induces relief in animal models of liver pathology [135, 136] and in patients with liver problems [137, 138]. MSCs can induce beneficial changes in minor populations of liver cells. For example, in a murine liver fibrosis model MSCs are able to induce a profibrotic to resolutive phenotype shift in hepatic macrophages, which is a key early event promoting fibrosis reversion [139].

MSCs display a number of additional useful properties including low immunogenicity, affinity to the injury sites, and the ability to modulate immune responses [134, 140]. They are also cheap in production. Unique MSC properties make them convenient cell material for the development of cell therapies, including cell therapies of liver diseases. For this reason the major part of preclinical and clinical research in the field of cell therapy of many human diseases is currently conducted with MSCs.

## 7. Liver Organ/Tissue Engineering

Orthotopic transplantation of allogeneic liver or its part is frequently the only life-saving option for patients with liver failure. Accordingly, reasonably priced and immune conflict-free donor liver substitutes are in urgent demand. There are chances that the problem of surrogate liver production can be solved by tissue engineering, an innovative strategy combining live cells with different natural and artificial materials to create transplantable functioning tissue or organ analogs (bioartificial grafts). While much more research is needed to adequately respond to organ transplantation challenges, bioengineered liver tissue is already used in drug metabolism studies [141].

Two liver engineering approaches have been experimentally checked to this point [142]. The first one involves repopulation of a decellularized liver stroma scaffold by hepatocytes, stem cells, endotheliocytes, and other cell types. Decellularization can be carried out by perfusion of the organ through its blood vessels with solutions containing enzymes and detergents [143]. It delivers an extracellular matrix scaffold free of cells and immunogenic proteins, reproducing in detail the architecture of liver stromal 3D backbone, offering support for repopulating cells and placing growing blood vessels and biliary ducts to their right positions. The ensuing scaffold can be recellularized in a bioreactor. In animal experiments repopulation of the scaffold can be carried out in vivo after heterotopic transplantation. Rat livers prepared utilizing decellularization/recellularization technology were successfully grafted into rats [144–146], while decellularized porcine liver scaffold populated with human cells was transplanted into a pig [147]. However, these and all subsequent publications reported grafted liver survival for less than a week underscoring the need for further technological improvements. Microencapsulation of hepatocytes before their engraftment into the tissue constructs facilitating long-time survival of functionally intact cells may be one of the useful improvements of the described technique [148].

Bioprinting is the second experimentally tested liver tissue engineering approach. Bioprinting combines 3D printing technology and biological materials including elements of extracellular matrix and live cells to form elaborate 3D tissue and organ structures, including vascular network. At present bioprinting already delivers metabolically active 3D hepatic tissue fragments which can be kept in a bioreactor alive and functioning for several days [149, 150].

Orthotopic transplantation of a recellularized liver graft to a patient with liver failure has not been performed yet and a number of problems need to be solved before clinical tests can be initiated. In addition to biotechnological issues, purely medical concerns, such as lack of a suitable surgical technique of orthotopic transplantation of engineered liver, need to be addressed. Choice of the cell material for liver engineering is also of great importance. As already indicated, autologous hepatocytes taken from patients with liver cirrhosis may be of poor quality. Allogeneic hepatocytes are difficult to get in large quantities and normally are immunologically incompatible with the graft recipient requiring life-long immunosuppression. Moreover, production of organs customized for each particular patient is a very costly and lengthy procedure. Recent basic research advances suggest the possibility of relatively cheap, essentially industrial production of organs for immunosuppression-free transplantation using immunologically matched allogeneic cells. The approach is based on the pluripotency induction technologies and the idea of the statistical prevalence of certain human leukocyte antigen (HLA) gene combinations within the population.

Immortal iPSC lines can be prepared from the majority of differentiated cell types including such easily accessible cells as skin fibroblasts and CD34 fraction of blood-borne cells. Like embryonic stem cells, generated pluripotent cells can be differentiated into virtually any cell type, including hepatocytes and other liver cells [107, 151]. Methods of hepatocytes production from iPSCs comprise several steps reproducing the stages of hepatocyte differentiation during ontogenesis. Three- and four-step protocols have been developed, each step guided by a specific set of growth and transcription factors. The four-step differentiation protocol was historically the first and included the stages of iPSC differentiation into the definitive endoderm, hepatocytic specification, hepatoblast expansion, and hepatocyte maturation [123]. Three-step protocols comprise consecutive iPSC differentiation into definitive endoderm, immature hepatocytes, and mature hepatocytes [124, 125]. Hepatocytes derived from iPSC have metabolic and functional characteristics similar to primary human hepatocytes [124, 152] and retain basic hepatocyte properties in bioartificial liver microenvironments [153].

Except hepatocytes, liver tissue contains other cells necessary to ensure its functions. They all can be differentiated from iPSCs, and some of them can be differentiated from LSPCs. The ideal composition of cell cocktail for liver scaffold population or bioprinting is not known. There are many more problems to solve before transplantation of engineered liver constructs can proceed to the clinical testing phase and much more preclinical research is needed [154].

Customized engineering of liver for a particular patient is going to be very expensive and large scale production of organs from allogeneic cells is the only option realistic from the economic point of view. Immunological distinctiveness of individual people is determined by two coexpressed haplotypes, represented by HLA-A, HLA-B, and HLA-DR gene each. Importantly, there are statistically preferable patterns, because combinations of these three genes proved to be not random. It has been calculated that it is possible to select a limited number of HLA homozygous donors with statistically prevalent HLA gene patterns matching most of heterozygous potential cell and organ transplant recipients [155]. According to the calculations, 30 homozygous iPSC lines derived from donors selected from 15,000 Japanese individuals match 82.2% of potential Japanese recipients, while 50 lines from donors chosen from 24,000 people provide immunocompatible material to 90.7% of recipients. This finding provided the idea of national or global iPSC line registries [156–158]. Those registries could provide cellular material for immunosuppression-free cell therapy and tissue engineering. Cells can be produced in very large quantities, making them much cheaper.

Embryonic stem cells and iPSCs are undifferentiated pluripotent cells tailored to convert into various differentiated cells. As indicated in the Transplantation of MSCs, in vitro cultures of MSCs contain another kind of pluripotent cells that can be differentiated into hepatocytes. Since MSCs are cells of mesodermal origin, while hepatocytes belong to endodermal lineage, hepatogenic differentiation of a subpopulation of MSCs has been named “transdifferentiation.” It is not yet clear if transdifferentiation of MSCs can yield fully functional hepatocytes, but research in this field is underway.

Cell phenotype and behavior strongly depend upon diverse biochemical signals, oxygen supply, mechanical load, topography of the immediate neighborhood, and many other parameters. The optimum values of those parameters are attainable in full only inside a healthy body. Full hepatogenic differentiation of iPSCs or other cells and normal functional activity of differentiated cells can be achieved only after orthotopic transplantation of bioengineered liver and the establishment of its regular connections with the recipient's organism through blood supply, lymph, innervation, and so forth. At present it is not yet clear what quality checks should be performed on engineered liver before transplantation.

## 8. Ongoing Clinical Trials of Cell-Based Therapies of Liver Diseases

Clinical trials of several cell therapy protocols registered at “https://www.clinicaltrials.gov/” site and aimed at treating liver diseases are underway, though they make just a small fraction of all ongoing clinical trials of cell therapy methods. Back in 2014 Moore and coauthors [159] were the first to review results of 33 studies selected by the following relatively strict criteria: median patients number 10 and follow-up for no less than 6 months. This was the first review covering this topic. On August 16, 2016, “Liver failure AND Cell therapy” inquiry revealed 9 registered trials (stage I/II), while “Cell therapy” inquiry revealed 794 trials (stages I/II or III). Interestingly, “Liver failure AND hepatocyte transplantation” query showed just 3 trials of which 1 was withdrawn and the 2 were suspended suggesting problems with deriving sufficient quantities of cells for transplantation or unsatisfactory efficacy. Moore's review and our search results demonstrate that regular clinical testing of cell therapies in hepatology is in its initial phase. Its progress strongly depends upon the results of the current trials.

It is already clear, though, that the methods of cell therapy can be further improved [160, 161]. Optimization strategies may include search for the most convenient source of cell material, comparing primary versus cultured cells versus cell lines, combining different cell types, experimenting with culture conditions:number of passages, variable oxygen supply, using genetically manipulated cells capable of induced growth factor release or enhanced in vivo differentiation, trying various administration routes, finding optimal cell numbers, best timing, and number of transplantations, enhancing engraftment of transplanted cells into the liver tissue, and their proliferation after engraftment.

## 9. Conclusion

Data examined in this review demonstrate that adult mammalian liver possesses considerable ability for homeostatic and injury-induced regeneration. The cellular mechanisms of liver regeneration are different from those in other organs in that restoration of the mass of liver parenchyma is achieved by compensatory hypertrophy and hyperplasia of the differentiated parenchymal cells, hepatocytes. Actually, hepatocytes (or a fraction of them) may be considered bipotent progenitor cells capable of dedifferentiation, rapid proliferation, and redifferentiation into new hepatocytes and cholangiocytes. In the majority of internal organs resident stem/progenitor cells play a major role in homeostatic regeneration of parenchyma and in the repair of damage caused by disease or trauma. In hepatic tissue their participation in homeostatic and injury-induced regeneration is still a matter of debate. Participation of blood-borne bone marrow stem cells in the replenishment of liver parenchyma has been proven, though the extent of their participation is limited. Experimental studies of liver regeneration provide valuable guidance to the management of patients with liver failure and to the development of new approaches, including methods of regenerative medicine, that is, cell therapy and tissue engineering.

Cell therapy with hepatocytes is directed to the replenishment of lost parenchymal cells. Transplanted hepatocytes induce paracrine effects as well. Transplantation of HSCs or MSCs promotes regeneration in many tissues throughout the body suggesting that paracrine signals produced by transplanted cells activate stem and certain differentiated cells of various tissues and organs in a similar way. At the cellular level those paracrine signals might induce stem cell proliferation, regulate mesenchymal-to-epithelial transition, suppress differentiation into fibrogenic myofibroblasts, and stimulate macrophage transition to anti-inflammatory phenotype. In addition, rare pluripotent cells present in HSC and MSC cultures may after transplantation differentiate into parenchymal cells of various tissues. All these effects of cell transplantation have been demonstrated in animal liver injury models.

Prospects of transplantation of engineered liver tissue, engineered liver lobe, or whole engineered organ seem good. Different approaches of liver tissue engineering are being developed and innovative tactics are being introduced, but it is not yet clear when clinical trials might begin.

In conclusion, it should be stressed once more that mammals, including humans, possess rather efficient mechanisms of liver regeneration that are activated spontaneously and can be further stimulated by cell therapy. In case of severe damage liver transplantation is the best option. Engineering of a transplantable liver or its part is an enormous challenge, but experimental studies show that it is probably doable.

## Figures and Tables

**Table 1:** Role of various cells in liver regeneration.

Type of cells	Animal model	Examples of similar human disease or state	Cellular mechanisms involved
Differentiated hepatocytes	Homeostatic regeneration, partial hepatectomy (rat, mouse), choline-deficient, ethionine-supplemented (CDE) diet (mouse), chronic CCl4 (mouse), diethyldithiocarbamate- (DDC-) induced liver damage (mouse), *α*-naphthylisothiocyanate- (ANIT-) induced liver damage (mouse)	Homeostatic regeneration, partial liver resection (cancer, bleeding after mechanical trauma, etc.), organ mass restoration after partial liver transplantation, liver fibrosis and cirrhosis, acute or chronic liver failure	Hypertrophy followed by hyperplasia and differentiation into hepatocytes or transdifferentiation into cholangiocytes; debated if all or a subpopulation (e.g., hybrid periportal hepatocytes) of hepatocytes participate

LSPCs	Liver poisoning by dipin, retrorsine, galactosamine (rat, mouse), CDE diet (mouse), chronic CCl4 (mouse), DDC-induced liver damage (mouse), ANIT-induced liver damage (mouse), and so forth	Acute or chronic liver failure, including liver tissue necrosis after poisoning or partial liver transplantation	Expansion and differentiation into hepatocytes, cholangiocytes, and probably other liver cell types; debated if this mechanism of liver regeneration exists

Blood-borne HSCs	Hepatocyte proliferation blockade combined with liver injury to induce oval cell proliferation after cross-sex bone marrow transplantation (rat, mouse, pig)	Any disease or state; liver transplantation	Transdifferentiation into hepatocytes; after liver transplantation transdifferentiated HSCs substitute donor hepatocytes; debated what subpopulation of HSCs (“endodermal progenitors,” multipotent precursors) participate; dusion with resident hepatocytes (?)

Blood-borne MSCs	Transplantation of human MSCs into immunodeficient partially hepatectomized immunodeficient mice, hepatocyte proliferation blockade combined with liver injury to induce oval cell proliferation after cross-sex bone marrow transplantation (rat, mouse, pig)	Any disease or state; liver transplantation	Transdifferentiation into hepatocytes and other liver cell types; after liver transplantation transdifferentiated MSCs substitute donor hepatocytes; debated what subpopulation of MSCs (Muse cells or others) participate; fusion with resident hepatocytes (?)
